# Structural Reorganization in Two Alfalfa Mitochondrial Genome Assemblies and Mitochondrial Evolution in *Medicago* Species

**DOI:** 10.3390/ijms242417334

**Published:** 2023-12-11

**Authors:** Xiaofan He, Xiaopeng Zhang, Yantian Deng, Rui Yang, Long-Xi Yu, Shangang Jia, Tiejun Zhang

**Affiliations:** 1 School of Grassland Science, Beijing Forestry University, Beijing 100083, China; hexf20@bjfu.edu.cn (X.H.); zxp1999@bjfu.edu.cn (X.Z.); dyantian@bjfu.edu.cn (Y.D.); yangrui0206@bjfu.edu.cn (R.Y.); 2 United States Department of Agriculture-Agricultural Research Service, Plant Germplasm Introduction and Testing Research, Prosser, WA 99350, USA; longxi.yu@usda.gov; 3 College of Grassland Science and Technology, China Agricultural University, Beijing 100193, China; shangang.jia@cau.edu.cn

**Keywords:** *Medicago sativa*, mitochondria, genome assembly, population evolution

## Abstract

Plant mitochondria are crucial for species evolution, phylogenetics, classification, and identification as maternal genetic material. However, the presence of numerous repetitive sequences, complex structures, and a low number of genes in the mitochondrial genome has hindered its complete assembly and related research endeavors. In this study, we assembled two mitochondrial genomes of alfalfa varieties of Zhongmu No.1 (299,123 bp) and Zhongmu No.4 (306,983 bp), based on a combination of PacBio, Illumina, and Hi-C sequences. The comparison of genome assemblies revealed that the same number of mitochondrial genes, including thirty-three protein-coding genes, sixteen tRNA genes, and three rRNA genes existed in the two varieties. Additionally, large fragments of repetitive sequences were found underlying frequent mitochondrial recombination events. We observed extensive transfer of mitochondrial fragments into the nuclear genome of Zhongmu No.4. Analysis of the *cox1* and *rrn18s* genes in 35 *Medicago* accessions revealed the presence of population-level deletions and substitutions in the *rrn18s* gene. We propose that mitochondrial structural reorganizations may contribute to alfalfa evolution.

## 1. Introduction

Plant mitochondria contain DNA independent of the nuclear genome, known as the mitochondrial genome, which tends to be larger in size in higher plants. The length of mitochondrial genomes varies significantly among plant species and can expand through the replication of its sequences, the transfer of nuclear genome fragments and chloroplast genome fragments [1,2,3]. To date, it has been reported that the mitochondrial genome of *Silene conica* is the largest among higher plants, with a size of 11,318 kb [4]. The mitochondrial genomes of *Medicago truncatula* and *Medicago polymorpha*, both belonging to the *Medicago* genus, have been assembled with lengths of 271,618 and 287,636 bp, respectively [5,6].

Unlike the relatively conserved characteristics of animal mitochondrial genomes, the mitochondrial genomes of higher plants have complex structures. In a study of mitochondrial DNA morphology in *Lactuca sativa*, it was found that mitochondrial DNA exists in a variety of molecular structures, such as branched linear, circular, linear, degraded, comet, and branched circular, which suggests that the mitochondrial genome does not exist in the common circular structure, but rather coexists in multiple forms [7]. Most studies have organized plant mitochondrial genomes into rings, which are defined as “primary rings”, because they contain all the genetic information of the mitochondria [8,9].

Higher plant mitochondrial genomes have low gene counts, highly repetitive sequences, and low gene densities, with intergenic regions accounting for approximately 90% of the entire genome [10]. Mitochondrial genomes can form heteroplasmies by integrating nuclear and chloroplast genome fragments and rearrangements mediated by repetitive sequences [11,12]. Studies have shown that the size of repetitive segments affects the frequency of genome recombination differently. Large repetitive sequences (>1000 bp) cause mitochondrial genome recombination more frequently than medium-sized repetitive sequences (100–1000 bp), whereas short repetitive sequences (<100 bp) have very little effect on recombination [13].

The advent of next-generation sequencing technologies has provided the ability to assemble complete mitochondrial genomes, particularly with the emergence of Nanopore sequencing technology, which can generate sequencing lengths of up to 2273 kb, providing molecular bases for a comprehensive understanding of mitochondrial genomes [14,15]. Compared to the nuclear genome, the mitochondrial and chloroplast genomes are much smaller, making them advantageous in sequencing and assembly. Therefore, mining evolutionary relationships from mitochondrial and chloroplast genomes is an alternative strategy [16]. In previous studies, we successfully assembled 35 chloroplast genomes of the *Medicago* genus and conducted evolutionary analyses of 55 *Medicago* accessions using published chloroplast genomes [17].

This study focused on two cultivated varieties of *M. sativa*, Zhongmu No.1 and Zhongmu No.4. We used both short and long reads to assemble their mitochondrial genomes and used Hi-C and third-generation sequencing data to validate the presence of heteroplasmy in both varieties. We analyzed and compared the differences between the mitochondrial genomes of Zhongmu No.1 and Zhongmu No.4, as well as their transfer to the nuclear genome or genomes of other species. Finally, using the Zhongmu No.4 mitochondrial genome as reference, we conducted an evolutionary analysis of *Medicago* accessions and found the genetic evidence that mitochondrial rearrangement and recombination contribute to alfalfa evolution.

## 2. Results

### 2.1. Sequencing and Assembly

In Zhongmu No.1, a total of 3,192,995,028 bp Illumina reads and 1,192,995,028 bp Nanopore reads were obtained, including 104,137,722 bp Illumina reads and 19,634,491 bp Nanopore reads derived from the mitochondria. The N50 value of the assembled genome was 1673 bp, and mitochondrial genome sequencing data represented approximately 2% of the total sequencing data.

For the analyses of Zhongmu No.4 PacBio whole-genome sequencing data downloaded from the CNCB database, 70,258,127,317 bp PacBio reads were obtained. From these reads, 145,101,882 bp of mitochondrial PacBio reads were isolated using seed sequences. The N50 value of the assembled genome was 25,631 bp, and the mitochondrial genome accounted for approximately 0.2% of the total sequencing data. Enriching the mitochondrial genome reads in the raw data through density gradient centrifugation improved the content of mitochondrial genome reads. However, the N50 length of the third-generation sequencing data was significantly reduced.

### 2.2. Characteristics of the Mitogenomes of Zhongmu No.1 and Zhongmu No.4

**Figure 1:**
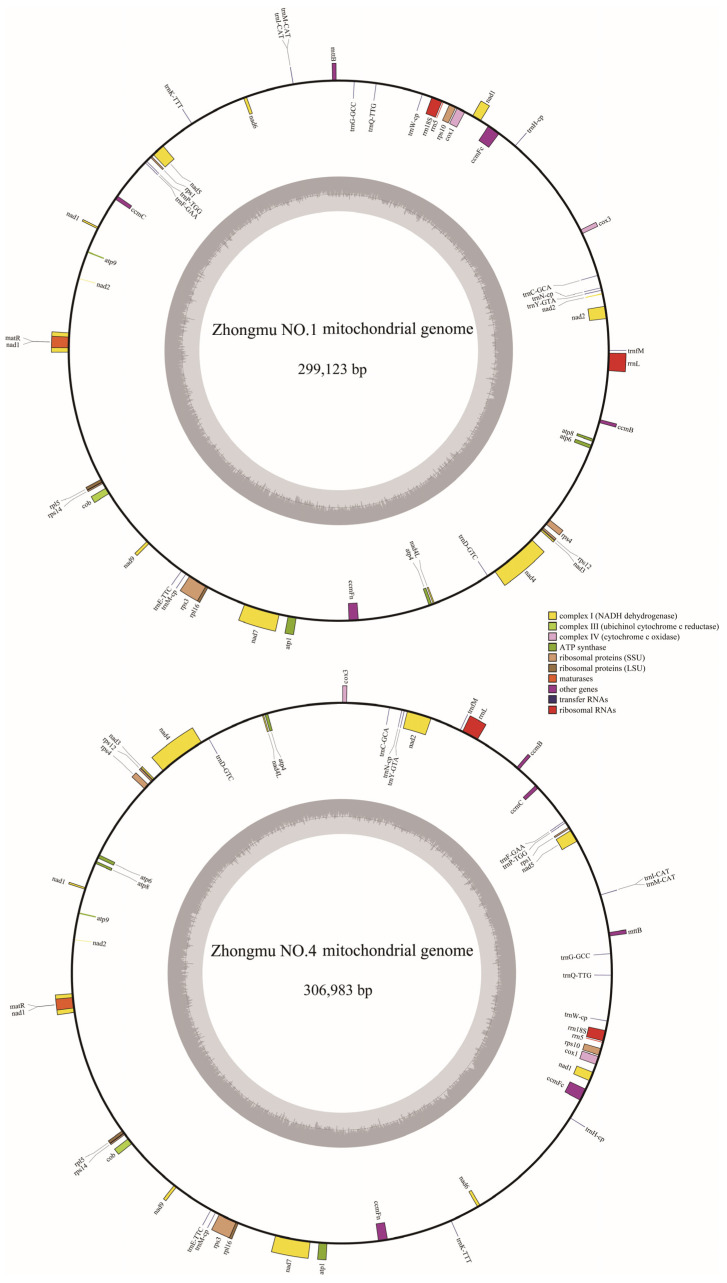
Circle maps of the mitochondrial genomes of Zhongmu No.1 and Zhongmu No.4.

The Zhongmu No.1 mitochondrial genome was assembled de novo using Illumina sequencing data. The average sequencing depth of the genome was 363×, with a size of 299,123 bp and a GC content of 45.35% (OR652280). The Zhongmu No.4 mitochondrial genome was assembled de novo using PacBio sequencing data. The sequencing depth of the genome was 268×, and the circular genome had a size of 306,983 bp and a GC content of 45.11% (OR652281). Through genome annotation, Zhongmu No.1 and Zhongmu No.4 were found to have the same number of mitochondrial genes, including thirty-three protein-coding genes, sixteen tRNA genes, and three rRNA genes (Figure 1).

The circular genome of Zhongmu No.1 consisted of nine contigs, named O1–O9 in descending order of sequence length. The circular genome of Zhongmu No.4 consisted of eight contigs, named F1–F8 (Figure S1). The repetitive sequences O7, O8, and F6 were larger than 1000 bp and were classified as large repeat sequences. The repeat sequences O9 and F8 were between 100–1000 bp in length and were classified as medium repeat sequences (Table S1).

### 2.3. Validation of Isoforms

**Figure 2:**
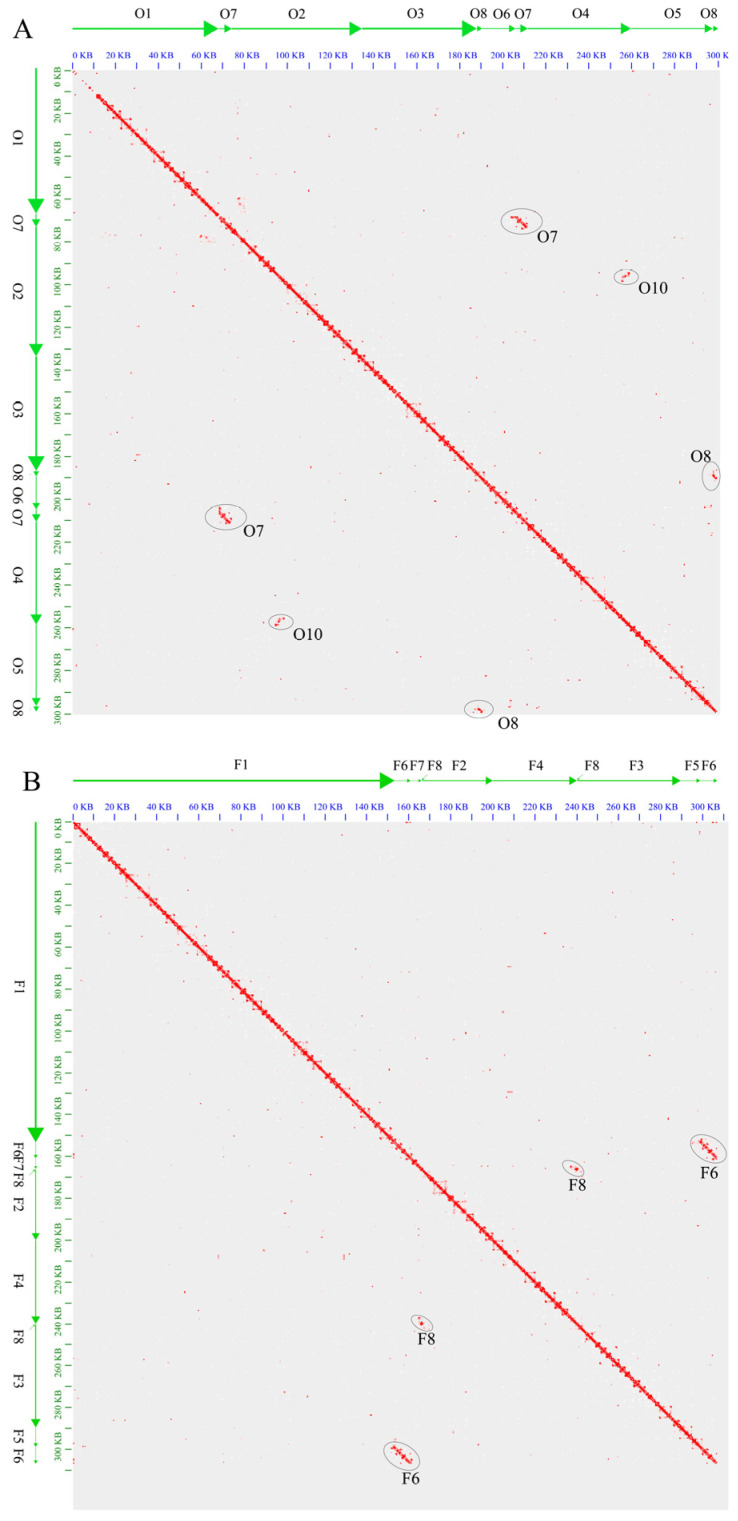
Long-distance analysis of mitochondrial genomes using Zhongmu No.4 Hi-C libraries. (**A**) Hi-C linkage plot of the Zhongmu No.1 mitochondrial genomes using the Hi-C libraries. (**B**) Hi-C linkage plot of Zhongmu No.4 mitochondrial genomes using the Hi-C libraries.

Hi-C libraries were used to validate the mitochondrial genome models derived from the PacBio reads in Zhongmu No.4. The distribution of repetitive F6 and F8 sequences on the Hi-C map indicated the presence of isoforms in the mitochondrial genome (Figure 2B). Four isoform configurations were validated using PacBio reads (Table 1). These results showed that the large repeat sequences F6 and F8 resulted in the formation of the Zhongmu No.4 mitochondrial genome.

Mapping reads from the Hi-C genomic library of Zhongmu No.4 to Zhongmu No.1 assembly revealed long-distance interactions. From the Hi-C map, it was inferred that O7 and O8 caused genome isoforms, whereas O9 did not lead to genome rearrangements (Figure 2A). Interestingly, another 200 bp segment was discovered on the Hi-C map, located at positions 95,737 bp-95,937 bp of O2 and 256,724 bp-256,925 bp of O4, which was named O10. Validation using Nanopore reads confirmed that repetitive sequences O7, O8, and O10 caused genome rearrangements (Table 1). Although the Nanopore data for the Zhongmu No.1 mitochondrial genome had a low sequencing depth and a short length, the isoforms formed by repetitive sequences O7, O8, and O10 were validated by at least one Nanopore read. On the other hand, O9 was shown not to cause genomic rearrangements (Table 1).

### 2.4. Evolution of the Mitochondrial Genomes of Zhongmu No.1 and Zhongmu No.4

**Figure 3:**
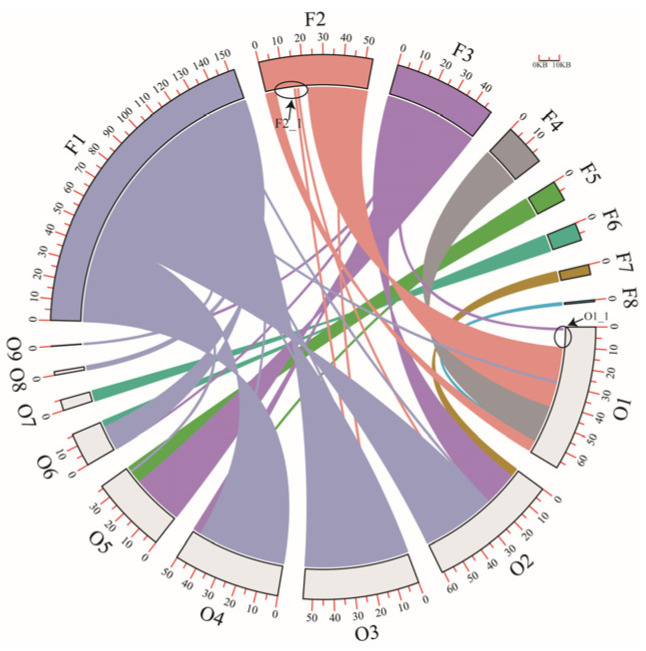
Visualization of homologous sequences between the Zhongmu No.1 and Zhongmu No.4 mitochondrial genomes. O1–O9, nine contigs comprising the Zhongmu No.1 mitochondrial genome. F1–F8, eight contigs comprising the Zhongmu No.4 mitochondrial genome.

Zhongmu No.1 and Zhongmu No.4 are two widely cultivated alfalfa varieties in northern China and are derived from different parental materials. In this study, we assembled and analyzed the mitochondrial genomes of Zhongmu No.1 and Zhongmu No.4, revealing differences in genome size between the two. A total of 288,114 bp were shared between the two genomes, accounting for 96.32% and 93.85% of the Zhongmu No.1 and Zhongmu No.4 mitochondrial genomes, respectively. Figure 2A shows an 11 kb gap in O1 and a 600 bp gap at the junction of O1 and O7. A co-linearity analysis comparing the contigs of Zhongmu No.1 and Zhongmu No.4 mitochondrial genomes identified unaligned segments in F2 and O1, named O1-1 and F2-1, respectively, indicating differences between the two genomes (Figure 3).

**Figure 4:**
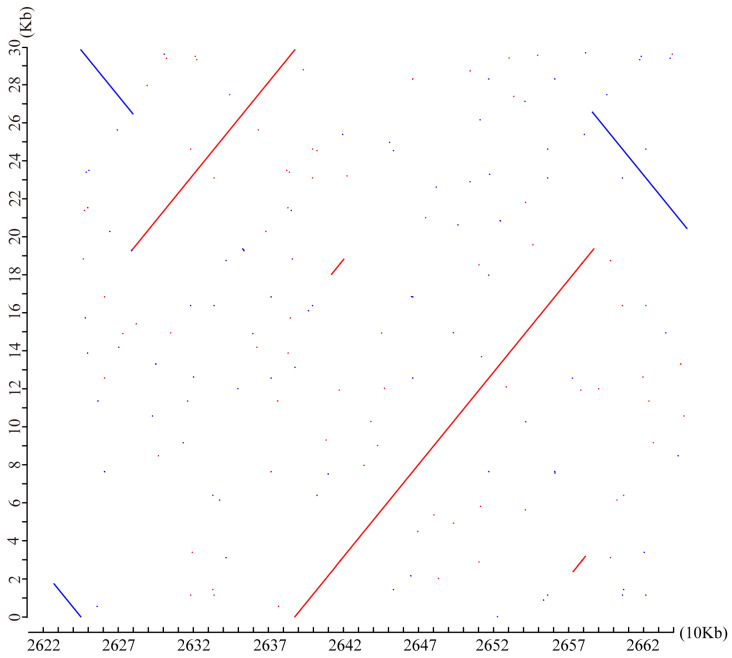
Distribution of mtDNA (y-axis) on nuclear chr_6_4 (26,220-26,620 kb, x-axis) in Zhongmu No.4. Red and blue indicate genome fragments on the positive and negative strands of the nuclear chromosomes, respectively.

To investigate whether these segments originated from the nuclear genome, we aligned the mitochondrial genomes of Zhongmu No.1 and Zhongmu No.4 with the nuclear genomes of Zhongmu No.1 and Zhongmu No.4. The results showed that O1-1 did not originate from the nuclear chromosome, whereas F2-1 aligned to chr6_2, chr6_3, and chr6_4 of the Zhongmu No.4 nuclear genome (Figures S2 and S3). Notably, the complete Zhongmu No.4 mitochondrial genome was present on chr6_4, spanning 26,220 kb to 26,620 kb (Figure 4). This phenomenon could be attributed to either the transfer of the mitochondrial genome to the nuclear genome or an assembly error that resulted in the assembly of the mitochondrial genome onto a nuclear chromosome.

**Figure 5:**
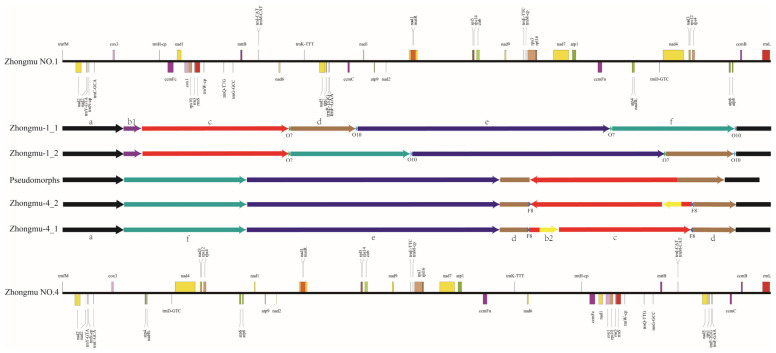
Interconversion between the mitochondrial genomes Zhongmu No.1 and Zhongmu No.4. Zhongmu-1_1 and Zhongmu-1_2 are isomers formed by recombination through the homologous repeat sequences O7 and O10. The pseudomorphic state is a possible isomer. Zhongmu-4_1 and Zhongmu-4_2 are isomers formed by recombination through the reverse repeat sequence F8.

Finally, we proposed a hypothesis for the evolution of the mitochondrial genomes from Zhongmu No.1 to Zhongmu No.4 (Figure 5). The Zhongmu-1_1 mitochondrial genome underwent rearrangements mediated by repetitive segments O7 and O10, forming segments d and f, and was named the Zhongmu-1_2 isoform. Segment d contained two sequences of 61 bp and 44 bp, causing segment c to be inserted in reverse orientation, forming an intermediate state; segment b1 was completely lost during this process. In the Zhongmu No.4 mitochondrial genome, the presence of the reverse repetitive sequence F8 led to the inversion of sequences c and b2, forming the isoform Zhongmu-4_2, followed by the loss of segment b2 to form an intermediate state. The four mitochondrial genomes, Zhongmu-1_1, Zhongmu-1_2, Zhongmu-4_1, and Zhongmu-4_2, were validated using the third-generation reads, with the intermediate state representing a hypothetical intermediate stage.

### 2.5. Evolutionary Analysis of the Medicago Accessions

**Figure 6:**
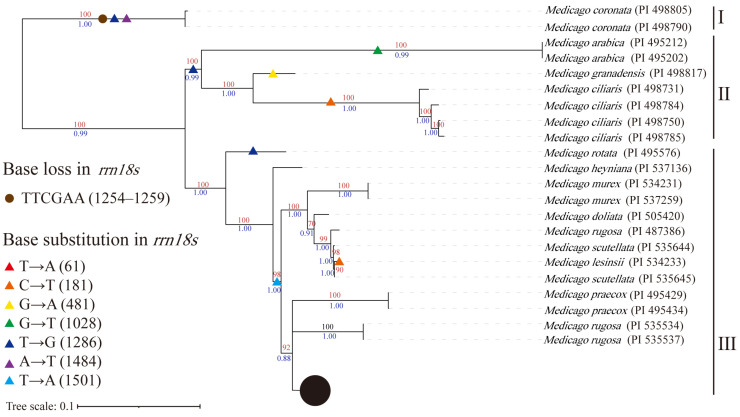
Phylogenetic relationships and *rrn18s* gene base substitutions in *Medicago* species. The maximum likelihood phylogenetic tree was estimated from SNPs with a maf >0.05 and a max missing rate <0.10. The red numbers represent the bootstrap of the ML phylogenetic tree, and the blue numbers represent the BI phylogenetic tree of the posterior probabilities.

Using the Zhongmu No.4 mitochondrial genome as a reference sequence, we performed SNP calling and filtering of the sequencing data of 35 *Medicago* accessions, resulting in 2559 SNPs. The maximum likelihood (ML) and Bayesian (BI) phylogenetic trees were constructed, resulting in phylogenetic trees with three clusters (Figure 6 and Figures S4 and S5). The BI and ML phylogenetic trees were identical.

Through sequencing data analyses, we assembled the conserved genes *cox1* and *rrn18s* in the mitochondrial genomes of the 35 *Medicago* accessions, using the alfalfa *cox1* and *rrn18s* genes as reference sequences. The *cox1* gene was highly conserved among the 35 *Medicago* accessions, with no insertions, deletions, or substitutions observed. However, the *rrn18s* gene exhibited nucleotide deletions and substitutions, with the absence of TTCGAA in cluster I. The T→G substitution at position 1286 was observed in clusters I, II, and III (*Medicago rotata*). The T→A (1501) substitution was also found in cluster III (excluding *M. rotata* and *Medicago heyniana*). The *rrn18s* gene of *M. heyniana* was identical to the *M. sativa* mitochondrial *rrn18s* gene, whereas nucleotide substitutions at other positions were specific to each species.

**Figure 7:**
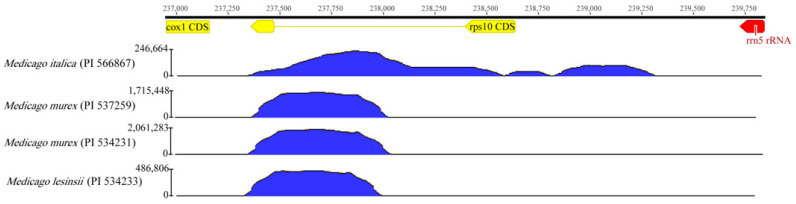
Read coverage comparison near mitochondrial *rps10* gene regions in the four *Medicago* accessions.

Furthermore, we observed abnormal sequencing depths in *Medicago italica* (PI 566867), *Medicago murex* (PI 566867), and *Medicago lesinsii* (PI 566867), with the highest sequencing depth reaching 2,061,283 near the *rps10* gene, far exceeding the average sequencing depth (Figure 7).

## 3. Discussion

### 3.1. Mitochondrial Genome Sequencing Strategies

Characterizing plant mitochondrial genomes has been challenging due to their complexity and frequent rearrangements. Previous studies have shown that plant mitochondrial genomes undergo frequent recombination events, leading to complex and noncircular structures [18]. To accurately determine the in vivo sequences and arrangements, it was necessary to use long reads at a high coverage depth, as paired-end reads alone do not capture recombination events involving longer repeats. In the mitochondrial genome assembly of *Agrostis stolonifera*, Li et al. directly sequenced the entire genome and filtered the mitochondrial genome sequences using seed sequences [19]. A purification step was performed before sequencing to avoid integrating mitochondrial sequences into the nuclear genome.

In this study, we enriched the Zhongmu No.1 mitochondrial genome using density gradient centrifugation and Illumina and Nanopore sequencing was performed. The results showed that the mitochondrial genome represented only 2% of the total sequencing data, indicating a low abundance of mitochondrial DNA. The N50 value of the Nanopore reads for Zhongmu No.1 was 1478 bp, which was shorter than the lengths typically obtained from third-generation sequencing. The difficulty in purifying mitochondria, the presence of incompletely developed chloroplasts, and the inability to eliminate nuclear DNA contributed to the low mitochondrial content and contamination with chloroplast genome reads.

### 3.2. Validation of Isoforms

Plant mitochondrial genomes are prone to rearrangements and exist in various isoforms due to their low gene density, high repeat content, and frequent recombination events mediated by repeats [20]. In *L. sativa*, more than 40 out of 98 fields of mitochondrial DNA exist in branched linear forms, whereas more than 20 out of 98 exist in circular forms [7]. This study used Hi-C technology to validate genome rearrangements and capture potential isoforms in the mitochondrial genome.

We observed that large repeat sequences (O7, O8, and F6) and an 836 bp reverse repeat sequence (F8) caused genome rearrangements, whereas a 135 bp repeat sequence (O9) did not lead to rearrangements. Interestingly, the number of each rearrangement type varied significantly. For example, the F7−, F8−, F4+, F2+, F8+, and F3− connections were six times more abundant in Zhongmu No.4 than in F7−, F8−, F2−, F4−, F8+, and F3−. However, the connections F1−, F6−, F1−, F5−, F6−, F7−, F6−, F1−, F6−, F5−, F6−, and F1− were represented similarly. This suggests that the probability of rearrangement mediated by repeat sequences is not equal for all types. There may be a correlation between repeat sequence length and the probability of isoform formation. Longer repeats display more similar probabilities for different isoforms and shorter repeats exhibit greater variation until an isoform is no longer formed. Additionally, Hi-C technology captured a 200 bp repeat sequence that was validated to cause genome rearrangements. Our results demonstrate that applying Hi-C technology enables a more comprehensive capture of mitochondrial genome isoforms, providing detailed insights into the evolutionary process of mitochondrial genome rearrangements.

### 3.3. Genomic Differences in Zhongmu No.1 and Zhongmu No.4

The mitochondrial genomes of higher plants can facilitate the exchange of genetic material between nuclear and organellar genomes at the gene level, as well as between different individuals. Kozik et al. elucidated the evolutionary process of the mitochondrial genomes of *L. sativa* and *Lactuca saligna* through genome assembly [7]. In this study, we assembled the mitochondrial genomes of two cultivated varieties of alfalfa, Zhongmu No.1 and Zhongmu No.4. Although both varieties have the same number of genes, they are different in length and structure. Fragment O1-1 could not be aligned with the genome, and only partial sequences were found in the NCBI database, leading to uncertainty regarding its origin. Fragment O1-1 may originate from the gap region of the nuclear genome. Fragment F2-1 was aligned with chromosomes chr6_3 and chr6_4 of the nuclear genome of Zhongmu No.4. These results suggest that the mitochondrial genome may integrate genetic material from the nuclear genome.

### 3.4. Population Evolution Analysis

Mitochondria and chloroplasts are organelles with a complete genetic system. Compared to the nuclear genome, they exhibit maternal inheritance and are more amenable to assembly. Therefore, organelle genomes are crucial in species evolution, phylogenetics, classification, and identification [21]. In a previous study, we conducted an evolutionary analysis of *Medicago* species using chloroplast genome data from 55 *Medicago* accessions [17]. The evolutionary trees derived from the chloroplast and nuclear genomes were identical, indicating that the plastid genome can serve as a substitute for the nuclear genome in interspecies evolutionary analysis.

In this study, we performed population SNP calling and constructed a maximum likelihood (ML) phylogenetic tree using the Zhongmu No.4 mitochondrial genome. Compared to the phylogenetic tree constructed using the chloroplast genome, there were difference in the clusters which contain different species, but the order of species divergence was largely consistent. This suggests that SNP information from the mitochondrial genome can reveal complete evolutionary relationships, but it is not as effective as using chloroplast-genome- and nuclear-genome-encoded proteins for species classification. Furthermore, we assembled the *cox1* and *rrn18s* genes in 35 *Medicago* accessions. The *cox1* gene was identical across all 35 accessions, with no base mutations. The *rrn18s* gene exhibited base mutations and deletions. The T→G (1286) mutation was widespread in early-diverging *Medicago* species, whereas the T→A (1501) mutation was widespread in later-diverging *Medicago* species. Base deletions were found only in the earliest diverging *Medicago coronata*. 

Chio et al. discovered a segment derived from the mitochondrial *rps10* gene that is widely inserted into the nuclear genome in *M. polymorpha*, naming it rps10-like [5]. We also observed this phenomenon in four accessions of *Medicago* in the present study.

## 4. Materials and Methods

### 4.1. Taxon Sampling, DNA Extraction, and Sequencing

In this study, we used the cultivated alfalfa variety Zhongmu No.1 (*M. sativa*), which was grown in the experimental field of the Hebei Academy of Agriculture Sciences in Langfang, China. Tender leaves and young roots of alfalfa were collected and stored at −80 °C for further use. Mitochondria from root and leaf tissues were enriched using Percoll gradient centrifugation, and the precipitate was stored at −20 °C [22]. Genomic DNA was extracted from the roots using the DNAsecure Plant kit (Catalog No. DP320-03, Beijing, China). A portion of the DNA was sequenced using the MinION platform (Oxford Nanopore Technologies, Oxford, UK). The remaining DNA was used to construct libraries for paired-end sequencing using the Illumina NovaSeq platform (Illumina, San Diego, CA, USA). 

Whole genome sequencing data of *M. sativa* accession Zhongmu No.4 were downloaded from the China National Center for Bioinformation (CNCB) with project number PRJCA004062. The data used in this study included PacBio data (CRR330258), Illumina data (CRR330261), and Hi-C data (CRR330262) [23]. The 35 *Medicago* accessions used in this study were previously described by Jiao et al. [13].

### 4.2. Mitochondrion Assembly and Annotation

Fastp v0.20.1 was used to assess the quality of the Illumina raw reads, whereas Nanoplot and NanoFilt were used to filter the Nanopore raw reads. Low-quality reads were removed from the data set [24,25]. The coding sequence (CDS) of the *M. truncatula* mitochondrial genome (NC_029641.1) was used as a reference to select the mitochondrial reads from the clean reads. The reference sequence of the *M. sativa* chloroplast genome (NC_042841.1) was used to remove the chloroplast reads from the mitochondrial reads [26]. Canu was used to correct the Nanopore and PacBio mitochondrial reads [27]. The de novo assembly of the plastomes was performed using Unicycler, Flye, and GetOrganelle v1.7.4.1 for further assembly improvement [28,29,30].

Geseq (https://chlorobox.mpimpgolm.mpg.de/geseq.html, accessed on 20 March 2023) and Annotator (PGA) were used for gene annotation. Protein-coding genes were compared with the National Center for Biotechnology Information (NCBI) using a BLAST search [31,32]. The start and stop codons of protein-coding genes, as well as the boundaries between introns and exons, were manually checked using the previously published mitochondrial genome of *M. truncatula* (NC_029641.1) as a reference.

### 4.3. Validation of Isoforms

Long-read sequencing data were used to validate the isoforms, and the genome structure was visualized using Bandage [33]. If the contig length was less than 1000 bp for repetitive contigs, we counted the number of sequencing reads that covered the entire repeat sequence. If the repeat sequence was larger than 1000 bp, we counted the number of sequencing reads spanning 250 bp on both sides of the junction point and the entire repeat sequence. BLAST was used as alignment software, with an e-value < 1 × 10^−5^ and an alignment length > 100 bp [34].

The HI-C library of Zhongmu No.4 was used to validate each repetitive sequence for isoform formation. Assembly 1 of Zhongmu No.1 and Zhongmu No.4 was used as the reference sequence for HI-C analysis. Juicer 1.6 was used to process the HI-C data, and Juicebox_1.11.08 was used to visualize the HI-C results [35].

### 4.4. Collinearity Analysis

The contigs of the Zhongmu No.1 and Zhongmu No.4 mitochondrial genomes were aligned using BLAST 2.13.0+, with an e-value < 1× 10^−5^ and a length > 100 bp. The results were visualized using TBTools–II [36,37].

To investigate the origin of the O1-1 and F2-1 sequences, we first aligned these two sequences with the “Zhongmu No.1” and “Zhongmu No.4” alfalfa genomes, respectively. We then searched for the sources of these two sequences in NCBI.

### 4.5. SNP Calling

Sequencing data from the 35 *Medicago* accessions were first filtered for quality using the Fastp program’s default parameters [24]. The reads from paired-end sequencing were then mapped to the genome of the Zhongmu No.4 mitochondrion using the BWA-MEM (BWA 0.7.17) default mapping options [38]. The resulting SAM data were converted into BAM files and sorted using the default parameters with SAMtools 1.18 [39]. Duplicate reads were marked using Picard Tools (http://broadinstitute.github.io/picard/, accessed on 29 May 2023), and possible misinterpreted indels for the SNPs were repaired using the Genome Analysis ToolKit (GATK4 4.4.0.0) [40,41]. The SNP data were further filtered using VCFtools 0.1.16 to obtain a missing rate of less than 10%, a minor allele frequency of 0.05, and a mean read depth greater than 20 [42].

### 4.6. Population Evolution Analysis

We used 2,559 SNPs to construct a maximum likelihood (ML) phylogenetic tree and a Bayesian (BI) phylogenetic tree. ModelTest 3.7 was used to evaluate nucleotide substitution models for the BI phylogenetic tree and the ML phylogenetic tree [43]. The ML phylogenetic tree was constructed using RAxML 8.2.13 with the GTRCAT nucleotide substitution model and a bootstrap value of 1000 [44]. The BI phylogenetic tree was constructed using MrBayes 3.2.7 [45]. The simulation was conducted for 10,000,000 generations for Markov chain Monte Carlo (MCMC) sampling, starting from a random tree. One tree was sampled every 1000 generations. The first 25% (2,500,000 generations) were discarded as “burn-in”, and the remaining trees were used to estimate the phylogenetic trees and to test posterior probabilities. The resulting tree was visualized and decorated using ITOL [46].

### 4.7. Assembly of the cox1 and rrn18s Genes

The *cox1* and *rrn18s* genes of Zhongmu No.4 were used as reference sequences. BWA-MEM was used to map the paired-end sequencing data of the 35 *Medicago* accessions from Illumina to the reference sequences. The SAM data were then transformed into BAM files and processed using SAMtools 1.18. Bedtools 2.31.1 was used to convert the BAM files into Fastq files. Unicycler 0.5.0 was used to assemble the *cox1* and *rrn18s* genes of the 35 *Medicago* accessions.

## Figures and Tables

**Table 1:** Verification of the repeated contig connection method.

O7	Reads	O8	Reads	O9	Reads	F6	Reads	F8	Reads
O1−, O7−	28	O3−, O8+	24	/	/	F1−, F6−	195	/	/
O7−, O2−	31	O8+, O6−	38	/	/	F5−, F6−	240	/	/
O6−, −O7	25	O5−, O8+	40	/	/	F6−, F1−	222	/	/
O7−, O4−	28	O8+, O1−	8	/	/	F6−, F7−	216	/	/
O1−, O7−, O2-	0	O3−, O8+, O6−	3	O2−, O9+, O3−	28	F1−, F6−, F1−	84	F7−, F8−, F2−	27
O1−, O7−, O4-	1	O3−, O8+, O1−	1	O4−, O9+, O5−	39	F6−, F1−, F6−	0	F4−, F8+, F3−	77
O6−, O7−, O2-	0	O5−, O8+, O6−	1	O2−, O9+, O5−	0	F5−, F6−, F1−	97	F7−, F8−, F4+	290
O6−, O7−, O4-	0	O5−, O8+, O1−	0	O4−, O9+, O3−	0	F5−, F6−, F7−	108	F2+, F8+, F3−	292

“+” and “−” denote the direction of the contigs.

## Data Availability

Upon reasonable request, the datasets used and/or analyzed in this study are available from the corresponding author.

## Supplementary Materials

The following supporting information can be downloaded at: https://www.mdpi.com/article/10.3390/ijms242417334/s1.
