# Supplementary material for: Structural Reorganization in Two Alfalfa Mitochondrial Genome Assemblies and Mitochondrial Evolution in Medicago Species
Source: Int J Mol Sci. 2023 Dec 11;24(24):17334. doi: 10.3390/ijms242417334 (PMC10743518; doi:10.3390/ijms242417334)
Supplement: Supplementary file 1 [file ijms-24-17334-s001.zip › ijms-2687216-supplementary.pdf]

Table S1. Contig names and lengths.

| Contig | Length(bp) | Contig | Length(bp) |
|---|---|---|---|
| O1 | 68195 | F1 | 152686 |
| O2 | 60869 | F2 | 53247 |
| O3 | 54341 | F3 | 48635 |
| O4 | 50200 | F4 | 19830 |
| O5 | 36146 | F5 | 9665 |
| O6 | 15712 | F6 | 8247 |
| O7 | 5031 | F7 | 4754 |
| O8 | 1664 | F8 | 836 |
| O9 | 135 | | |

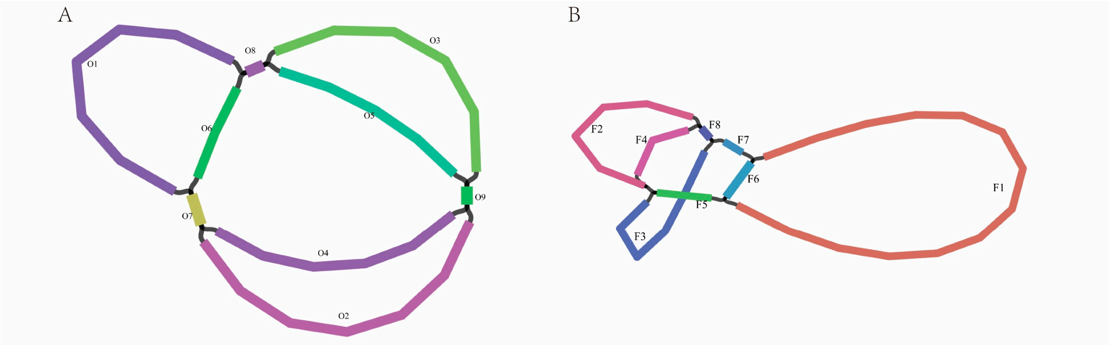


Figure S1. Mitochondrial genome bandage map. A. Zhongmu NO.1 mitochondrial genome. B. Zhongmu NO.4 mitochondrial genome.

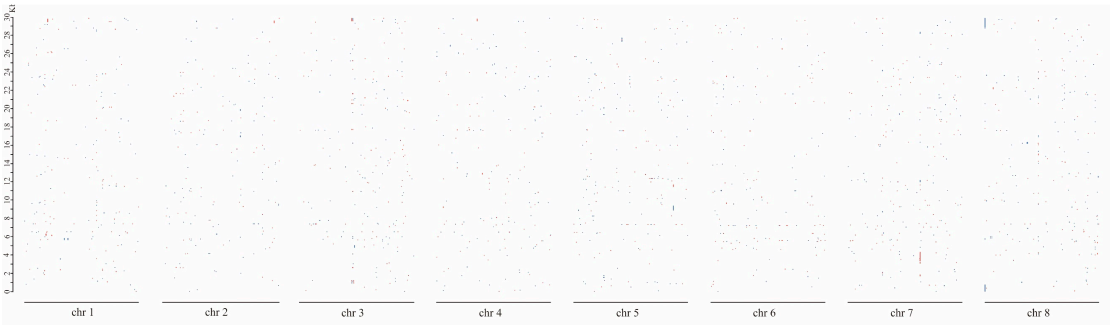


Figure S2. Distribution of mtDNA fragments on the nuclear chromosomes of *Medicago sativa*. The mtDNA fragments were plotted against the eight nuclear chromosomes of the *M. sativa* cultivar 'Zhongmu No.1' (x-axis) and Zhongmu NO.1 mitochondrial genome (y-axis). The red and blue colors indicate nupDNA fragments on the positive and negative strands of the nuclear chromosomes, respectively.

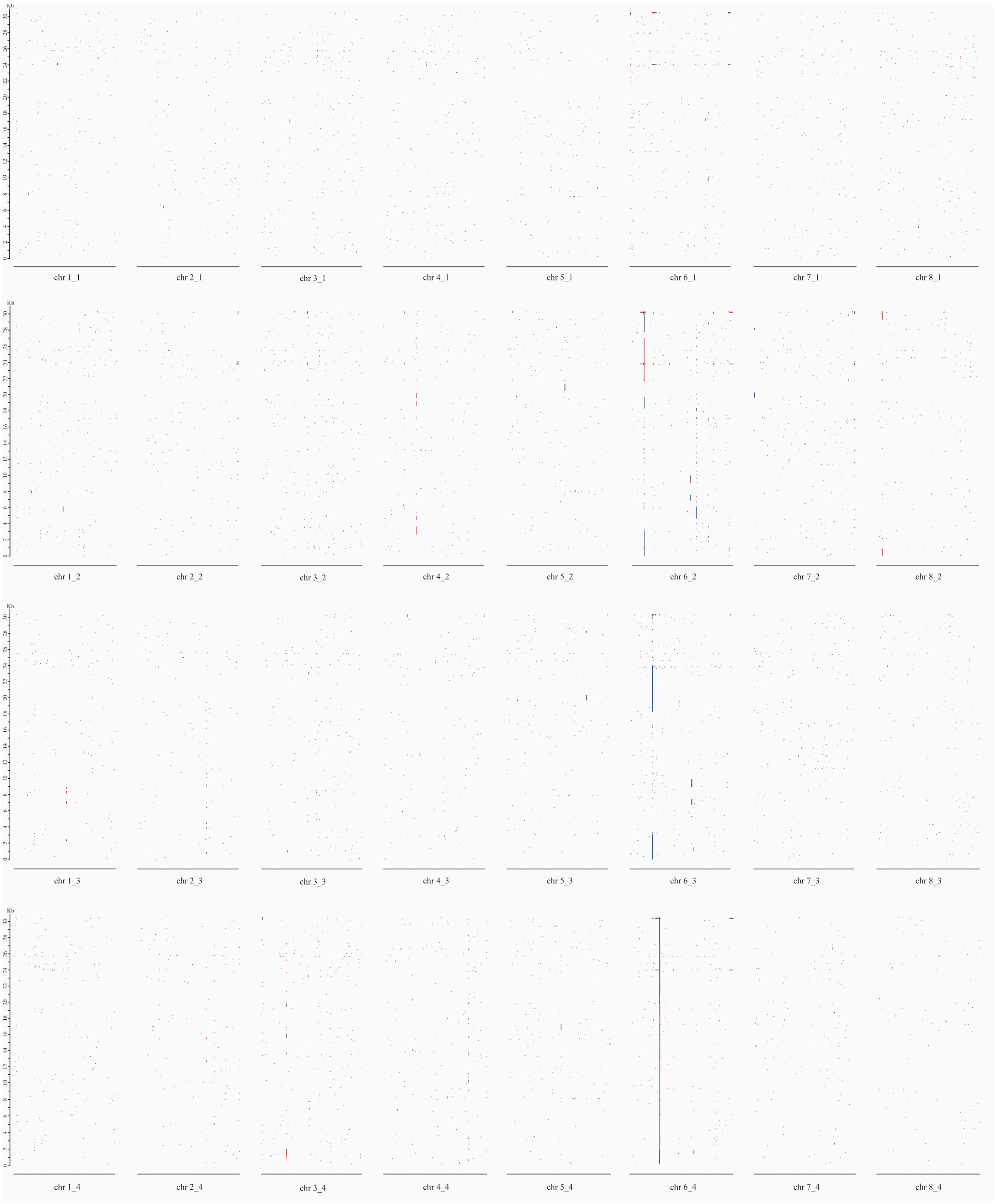


Figure S3. Distribution of mtDNA fragments on the nuclear chromosomes of *Medicago sativa*. The mtDNA fragments were plotted against the Thirty-two nuclear chromosomes of the *M. sativa* cultivar 'Zhongmu No.4' (x-axis) and Zhongmu NO.4 mitochondrial genome (y-axis). The red and blue colors indicate mtDNA fragments on the positive and negative strands of the nuclear chromosomes, respectively.

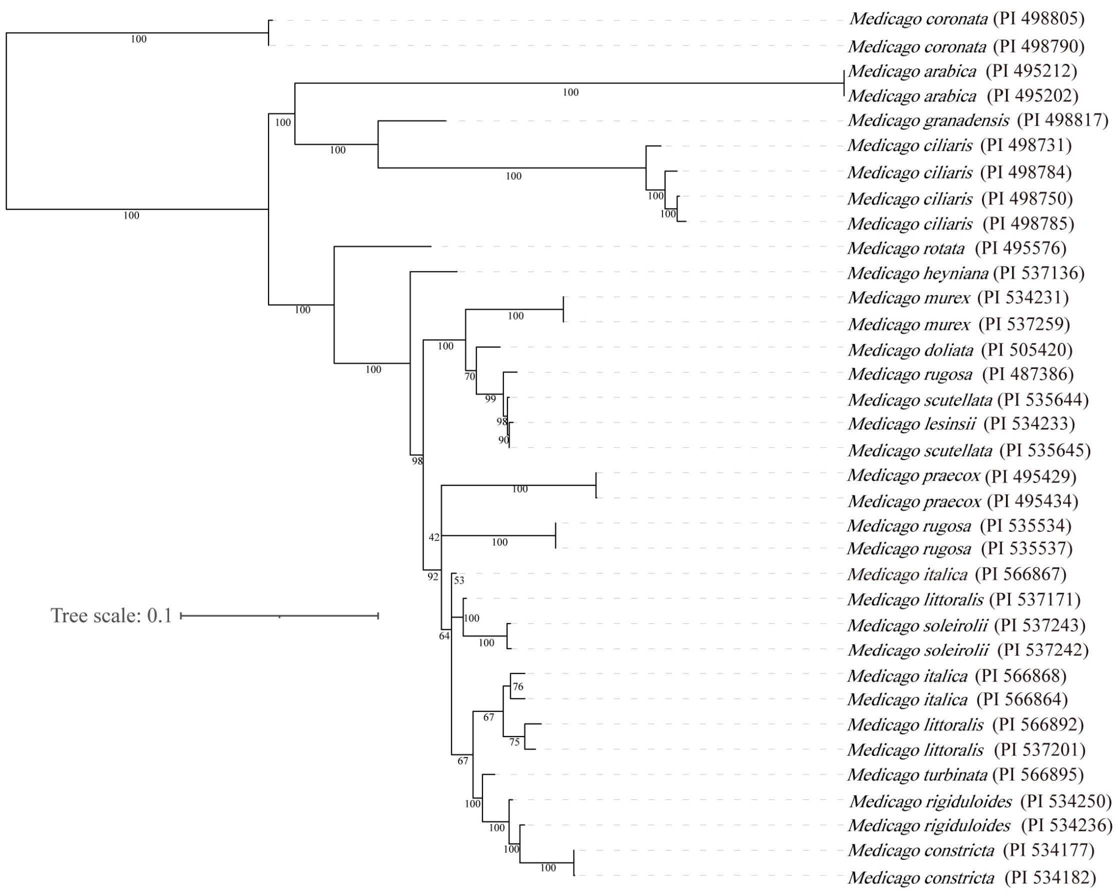


Figure S4. Maximum likelihood phylogenetic tree with bootstrap values.

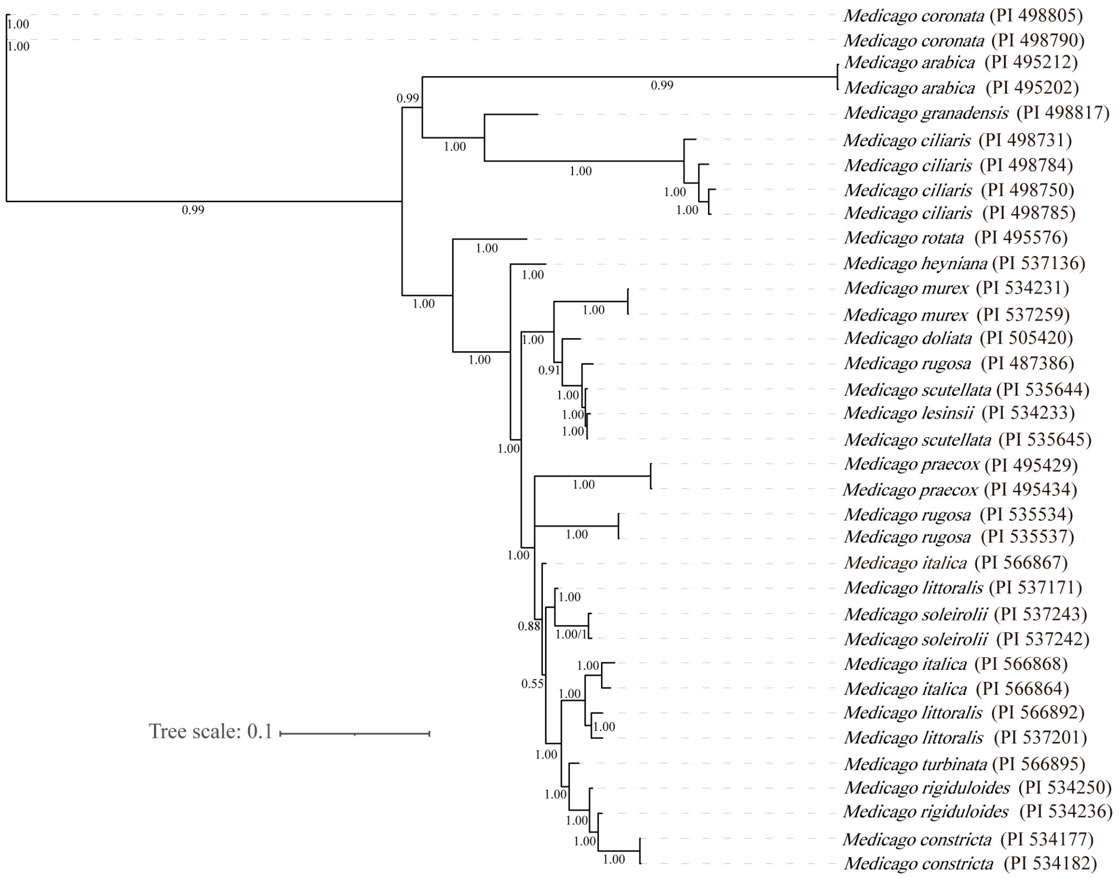


Figure S5. Bayesian phylogenetic tree with posterior probabilities.
